# Analysis of *eEF1A2* gene expression and copy number in cervical carcinoma

**DOI:** 10.1097/MD.0000000000032559

**Published:** 2023-01-13

**Authors:** Weinan Zheng, Fuyuan Jin, Fang Wang, Luyue Wang, Shaowei Fu, Zemin Pan, Haichen Long

**Affiliations:** a Department of Human Anatomy, Histology and Embryology, Chengdu Medical College, Chengdu City, Sichuan Province, China; b Department of Biochemistry and Molecular Biology, School of Medicine, Shihezi University, Shihezi City, Xinjiang Province, China; c Department of Biochemistry and Molecular Biology, Shihezi University School of Medicine (Branch College in Tarim University), Tarim University, Alaer City, Xinjiang Province, China.

**Keywords:** cervical cancer, *eEF1A2* gene, gene copy number, gene expression

## Abstract

To explore and analyze the expression of eukaryotic translation elongation factor 1 alpha 2 (*eEF1A2*) gene in cervical cancer tissues, its relationship with patient survival, gene mutations, and changes in copy number in cervical cancer and chronic cervicitis tissues.

**Methods:**

The expression of the *eEF1A2* gene in cervical cancer and its relationship with patient survival were analyzed using gene expression profile interactive analysis. Changes in *eEF1A2* expression in cervical cancer tissues were analyzed using cBioPortal, a portal for cancer genomics analysis. The *eEF1A2* copy number in cervical cancer tissues and chronic cervicitis tissues was determined by real-time fluorescence quantitative polymerase chain reaction. The relationship between the expression of *eEF1A2* protein and the clinical stage, pathological grade, and patient survival of cervical cancer was analyzed by the database: The Human Protein Atlas, an integrated repository portal for tumor-immune system interactions.

**Results:**

Gene expression profile interactive analysis database analysis showed no significant differences in the expression of *eEF1A2* between cervical cancer and normal cervical tissues (*P* > .05). The *eEF1A2* gene expression level was not correlated with the survival of cervical cancer patients (*P* > .05). Analysis of the cBioPortal database showed that 18 of 297 cervical cancer patients had *eEF1A2* gene changes, including missense mutation, splice mutation, amplification, and messenger RNA increase. There was no significant difference in *eEF1A2* gene copy number between cervical cancer and chronic cervicitis (*P* > .05). The Human Protein Atlas and an integrated repository portal for tumor-immune system interactions database analysis of immunohistochemical data showed that *eEF1A2* protein expression was no significant difference in clinical stage, pathological grade and patient survival of cervical cancer (*P* > .05).

**Conclusion:**

The *eEF1A2* gene was mutated in cervical cancer tissues. The *eEF1A2* gene copy number was not associated with changes in the expression of the *eEF1A2* gene in cervical cancer tissues.

## 1. Introduction

Cervical cancer is one of the most common malignancies of the female reproductive system worldwide,^[1]^ posing a serious threat to women’s health. Cervical cancer ranks second in incidence among female malignancies, second only to breast cancer, and its incidence has increased in recent years. According to the latest data released by the International Agency for Research on Cancer, in 2018, there were 5,69,847 new cases of cervical cancer worldwide and 3,11,365 deaths.^[2]^ Cervical cancer is the main cause of female death around the world, especially in developing countries. Eukaryotic translation elongation factor 1A (*eEF1A*) is a key factor involved in protein biosynthesis. The GTP-bound form of eEF1A forms a ternary complex with an acyl transfer RNA that delivers charged transfer RNA to the A site of the translation ribosome.^[3]^ The expression of eukaryotic translation elongation factor 1 alpha 2 (*eEF1A2*) interacts with zinc finger protein 1, and then associates with a group of receptors with tyrosine kinase ability to transmit signals from the cytoplasm to the nucleus, thereby promoting cell proliferation.^[4]^ This study analyzed the expression of the *eEF1A2* gene in cervical cancer tissues, its relationship with patient survival and gene mutation, and the effect of *eEF1A2* gene copy number changes on gene expression changes in cervical cancer tissues.

## 2. Methods

This is retrospectively research. All tissue were collected from the First Affiliated Hospital of Shihezi University School of Medicine tissue bank and the First People’s Hospital of Kashgar Prefecture tissue bank, Xinjiang. Ethics committee of the First Affiliated Hospital of Shihezi University School of Medicine approval it. Approval Number 2019-018-01.

### 2.1. Data sources

#### 2.1.1. Gene expression profile interactive analysis (GEPIA) database.

The GEPIA database is a database for interactive analysis of gene expression profiles based on tumor and normal samples in the cancer genome atlas program and the genotype-tissue expression project databases.^[5]^ In this study, the GEPIA database was used to analyze the expression of the *eEF1A2* gene in tumor tissues (including cervical cancer tissue) and its relationship with patient survival.

#### 2.1.2. Cancer genomics analysis web portal cBioPortal.

The cBioPortal is an open website for interactive exploration of multiple cancer genomics data sets.^[6]^ In this study, this website was used to analyze the genetic changes of *eEF1A2* in cervical cancer tissues.

### 2.2. Sample collection

Between June 2012 and December 2018, tissue specimens of 30 patients with cervical cancer and 30 patients with chronic cervical inflammation were collected from the First Affiliated Hospital of Shihezi University School of Medicine and the First People’s Hospital of Kashgar Prefecture, Xinjiang. All patients gave informed consent and were approved by the ethics committee of the First Affiliated Hospital of Shihezi University School of Medicine. The collected cervical tissue samples were cleaned with normal saline and stored in a refrigerator at -80°C. DNA was extracted from cervical cancer and chronic cervicitis tissues. A total of 30 cervical cancer tissue DNA samples, 22 chronic cervicitis tissue DNA samples, and one normal cervical tissue DNA sample were analyzed by 0.7% agarose gel electrophoresis, which showed that the DNA was not degraded and could be used for *eEF1A2* gene copy number analysis in cervical cancer and chronic cervicitis tissues.

### 2.3. Real-time fluorescence quantitative polymerase chain reaction (PCR) detection of *eEF1A2* gene copy number

#### 2.3.1. Primer design.

According to the full-length sequences of the internal reference gene RPP14 and the target gene eEF1A2 provided by the GenBank database on the NCBI website, the primers were designed with Primer 5 software and synthesized by Shanghai Tianhao Biological Engineering Co., Ltd. (Table 1).

**Table 1:** qRT-PCR primer information.

Gene name	Primer sequence (5*'*–3*'*)	Primer amplification region
*eEF1A2*-CNV	F:5*'*-GTCCTTCACCGACACGTTCTT-3*'*	GRCh38
	R:5*'*-CTGAGGTGAAGTCAGTGGAGATG-3*'*	chr20:63490551-63490648
*RPP14*-CNV	F:5*'*-GTCCTTGCGGCGAATAGGAGT-3*'*	GRCh38
	R:5*'*-CTGAGACGGCTCCCAGACAAC-3*'*	chr3:58306362-58306466

Note: F: forward primer, R: reverse primer.

*eEF1A2* = the expression of eukaryotic translation elongation factor 1 alpha 2, PCR = polymerase chain reaction.

#### 2.3.2. Real-time quantitative fluorescence PCR.

The SYBR® Green PCR Kit (Qiagen, Germany) was used for real-time quantitative PCR, and the RPP14 gene was used as an internal reference to detect the expression of eEF1A2. The experiment was repeated three times for each sample.

(i) Reaction system:
SYBR Premix Ex Taq (2×)---5 *µ*L
ROX Reference Dye (50×)---0.2 *µ*L
Primer (2 *µ*M)-------------------2 *µ*L
Template (DNA)--------------1.5 *µ*L
DdH2O--------------------------1.3 *µ*L
_____________________________________
Total volume--------------------10 *µ*L
(ii) Reaction conditions:
95°C pre-denaturation 30 seconds
95°C denaturation 5 seconds
55°C annealing 30 seconds } 40 cycles
72°C extension 30 seconds

### 2.4. Immunohistochemical (IHC) database analysis

Through the analysis of IHC data of the database: The Human Protein Atlas and an integrated repository portal for tumor-immune system interactions, the relationship between *eEF1A2* protein expression and clinical stage, pathological grade and patient survival of cervical cancer.

### 2.5. Statistical analysis

SPSS 19.0 software was used for all statistical analyses. Variance analysis was used for real-time fluorescence quantitative detection of *eEF1A2* gene copy number, and the obtained quantitative data represented at least three independent experiments. *P* < .05 indicated that the difference was statistically significant.

## 3. Results

### 3.1. Expression of the *eEF1A2* gene in tumor samples and matched normal tissues

**Figure 1.**
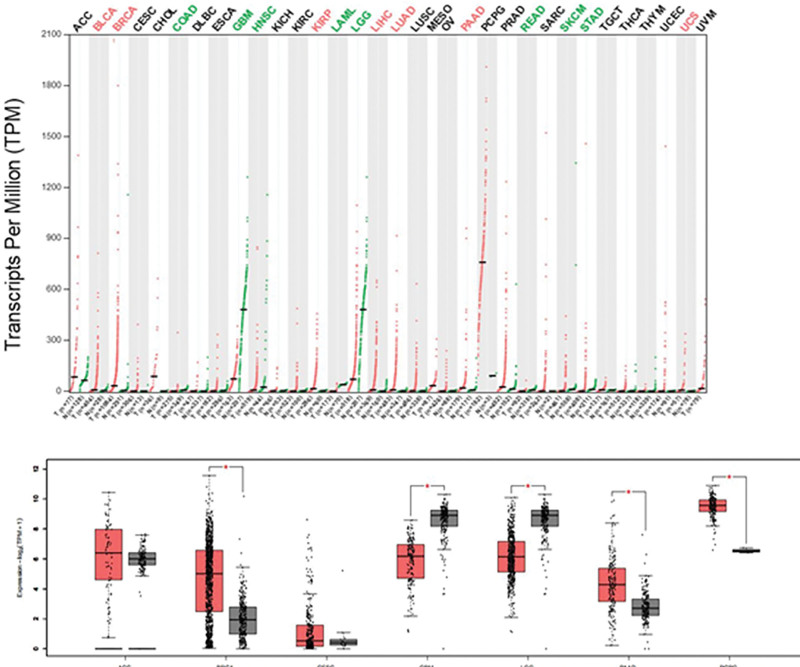
Expression of the *eEF1A2* gene in multiple tumors according to the GEPIA database. Note: (A) Gene expression profile of *eEF1A2* gene in a variety of tumors; (B) Expression level of the *eEF1A2* gene in ACC, BRCA, CESC, GBM, LGG, PAAD, and PCPG; *P < .05. BRCA = breast invasive carcinoma, CESC = cervical squamous cell carcinoma and endocervical adenocarcinoma, *eEF1A2* = the expression of eukaryotic translation elongation factor 1 alpha 2, GBM = glioblastoma multiforme, GEPIA = gene expression profile interactive analysis, LGG = lower grade glioma, PAAD = pancreatic adenocarcinoma, PCPG = pheochromocytoma and paraganglioma.

The GEPIA database was used to analyze the expression of *eEF1A2* in various tumors, including cervical cancer, and in normal samples. The results showed that the *eEF1A2* gene was highly expressed in breast invasive carcinoma, pancreatic adenocarcinoma, and pheochromocytoma and paraganglioma tissues, and was expressed at low levels in glioblastoma multiforme, brain lower grade glioma, and other tissues (Fig. 1).

### 3.2. Relationship between the expression of *eEF1A2* gene in cervical cancer tissues and cervical cancer patient survival and gene changes

**Figure 2.**
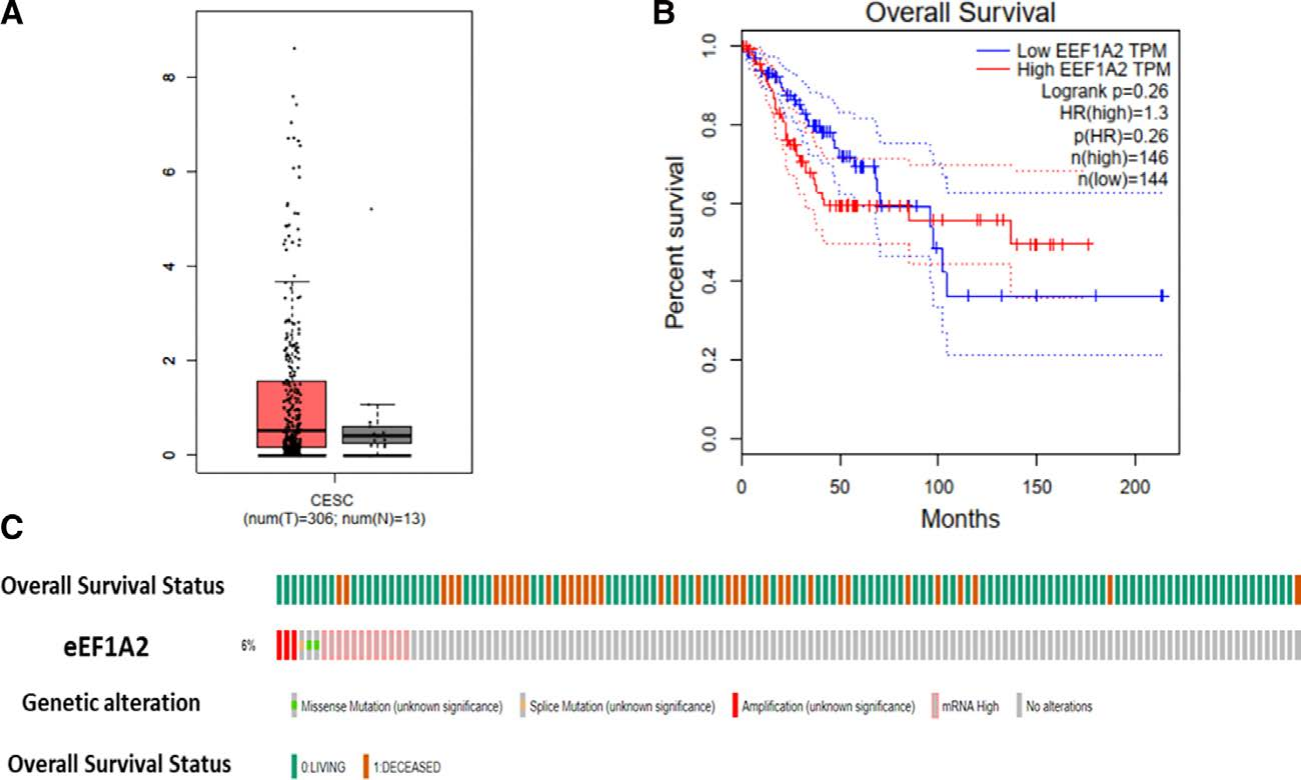
Expression of the *eEF1A2* gene and its relationship with patient survival and changes in gene expression in cervical cancer from bioinformatics database. Note: (A) Expression level of the *eEF1A2* gene in cervical cancer tissue; (B) relationship between *eEF1A2* gene expression and cervical cancer patient survival; (C) genetic changes of *eEF1A2* gene in cervical cancer tissue. *eEF1A2* = the expression of eukaryotic translation elongation factor 1 alpha 2.

The GEPIA database was used to analyze the expression of the *eEF1A2* gene in cervical cancer and its relationship with patient survival. The expression of *eEF1A2* did not differ significantly between cervical cancer and normal cervical tissues (*P* > .05, Fig. 2A); the *eEF1A2* gene was not associated with the survival of cervical cancer patients (*P* > .05, Fig. 2B). Analysis of the genetic changes of *eEF1A2* in cervical cancer tissues using cBioPortal showed that among 297 cervical cancer patients analyzed, 18 showed *eEF1A2* gene changes, including missense mutations, splice mutations, amplifications, and messenger RNA increase (Fig. 2C).

### 3.3. Detection of *eEF1A2* gene copy number in cervical cancer and chronic cervicitis tissues

**Figure 3.**
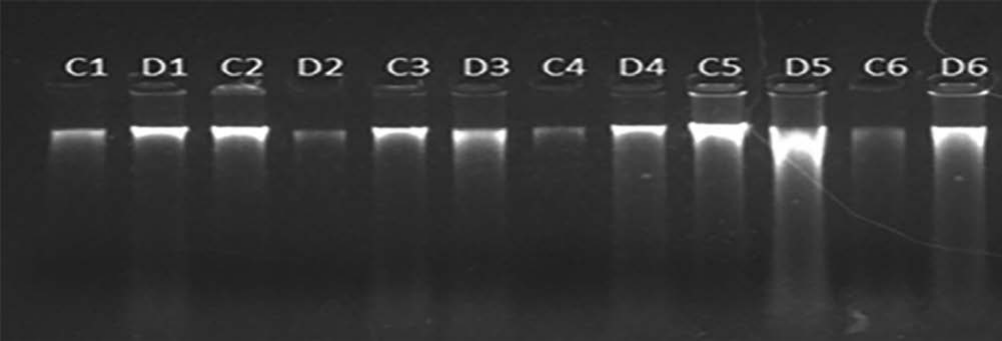
Results of 0.7% agarose gel electrophoresis of DNA in cervical tissue.

In preliminary work, we performed immunohistochemistry experiments and found that the expression level of the *eEF1A2* protein was significantly higher in cervical cancer tissue than in chronic cervicitis tissue. DNA was extracted from cervical tissue using a DNA extraction kit, and the quality of the extracted DNA was detected by 0.7% agarose gel electrophoresis. Complete DNA bands were detected in 30 cervical cancer tissue samples, 22 chronic cervicitis tissue samples, and 1 normal cervical tissue sample, indicating that the DNA was not degraded and the amount and quality of DNA were sufficient (*C*1, *D*1, *C*2, *D*2, *C*3, *D*3, *C*4, *D*4, *C*5, *D*5, *C*6, and *D*6 represent DNA electrophoresis bands of cervical cancer tissue and chronic cervicitis tissue). These tissue samples were used for real-time fluorescent quantitative PCR to detect *eEF1A2* gene copy number (Fig. 3).

**Figure 4.**
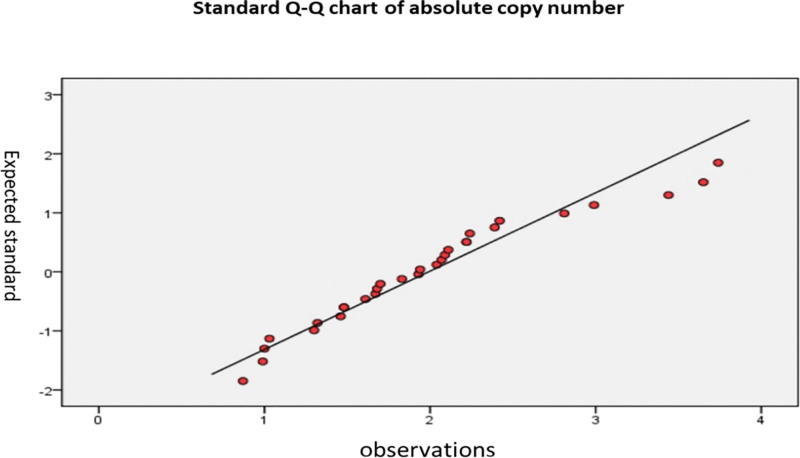
Absolute copy number in cervical cancer tissue.

**Figure 5.**
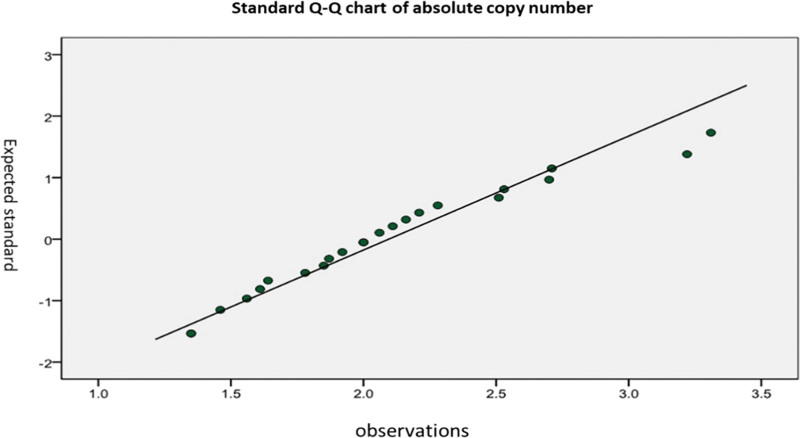
Absolute copy number in chronic cervicitis and normal tissues.

The absolute copy numbers of cervical cancer tissues and non-cancerous tissues detected by real-time fluorescent quantitative PCR are shown in Figures 4 and 5.

**Figure 6.**
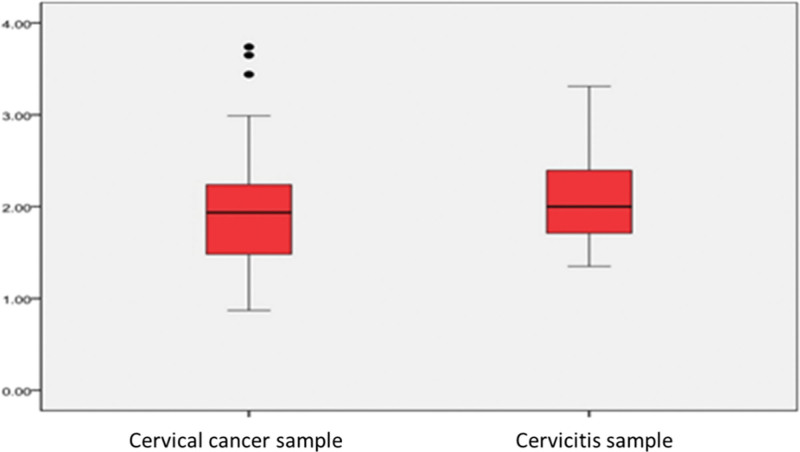
Variance analysis of 30 cases of cervical cancer and noncancer tissue copy number changes.

Variance analysis of 30 cervical cancer, 22 chronic cervicitis, and one normal cervical tissue samples showed no significant difference in the copy number of the *eEF1A2* gene between cancer tissues and non-cancerous tissues. These results indicated that the changes in the expression level of the *eEF1A2* gene in cervical cancer tissue may not be caused by changes in *eEF1A2* gene copy number (Fig. 6).

### 3.4. The relationship between the expression of *eEF1A2* protein and the clinical stage, pathological grade and survival of patients with cervical cancer

**Figure 7.**
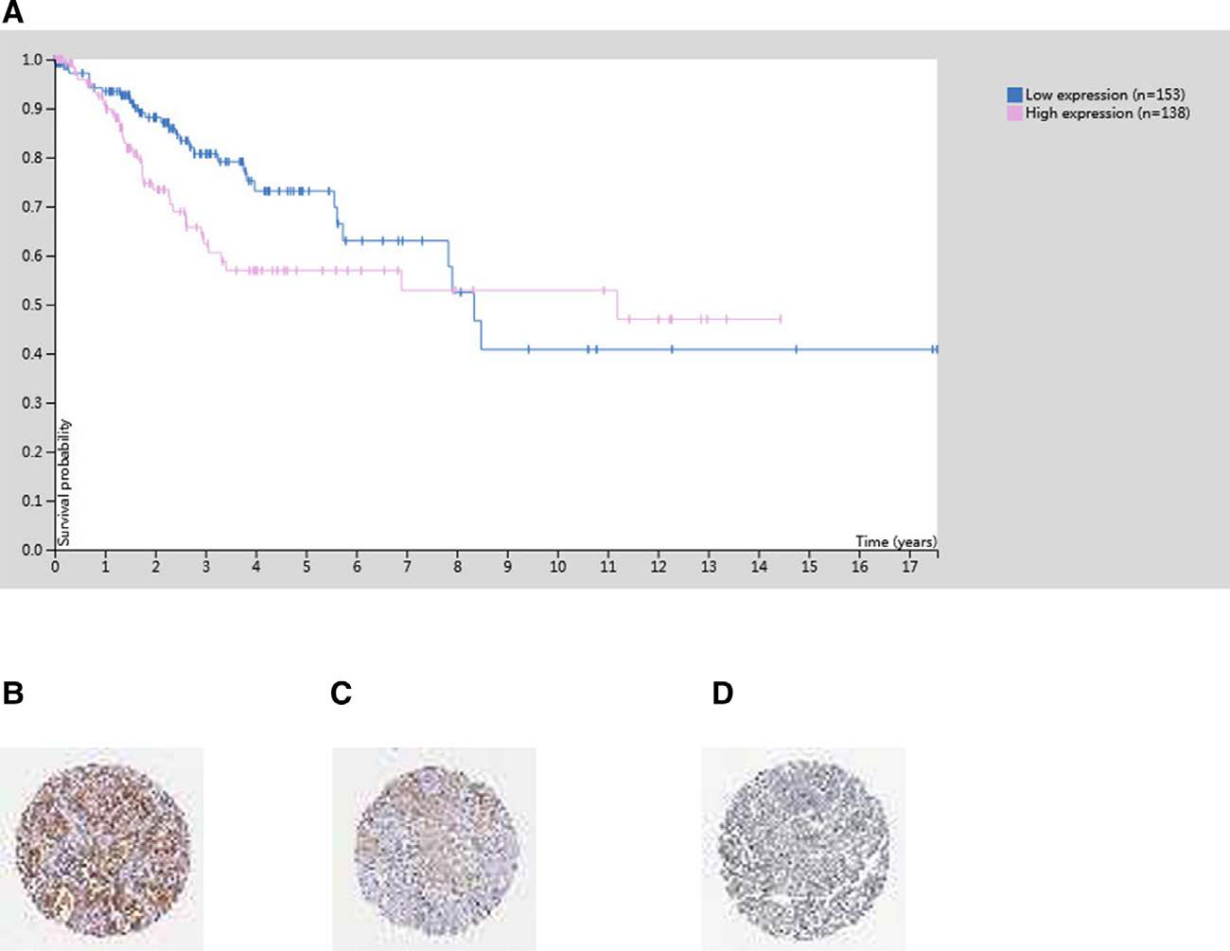
The human protein atlas database analysis. IHC, The relationship between the expression of *eEF1A2* and the survival of patients with cervical cancer. Note: (A) Female (n = 291), Dead (n = 71), Alive (n = 220), Low expression (n = 153), High expression (n = 138), There was no statistical significance between the expression of *eEF1A2* and the survival of cervical cancer patients. *P* = .068. (B) IHC High expression of *eEF1A2* protein in cervical cancer. (C) IHC Medium expression of *eEF1A2* protein in cervical cancer. (D) IHC Low expression of *eEF1A2* protein in cervical cancer. *eEF1A2* = the expression of eukaryotic translation elongation factor 1 alpha 2, IHC = immunohistochemistry.

Analysis of the relationship between the expression of *eEF1A2* in cervical cancer and the survival of patients by the database of The Human Protein Atlas. Female (n = 291), Dead (n = 71), Alive (n = 220), Low expression (n = 153), High expression (n = 138), *P* = .068 (Fig. 7A). IHC *eEF1A2* protein expression in cervical cancer (Fig. 7B, C, D).

**Figure 8.**
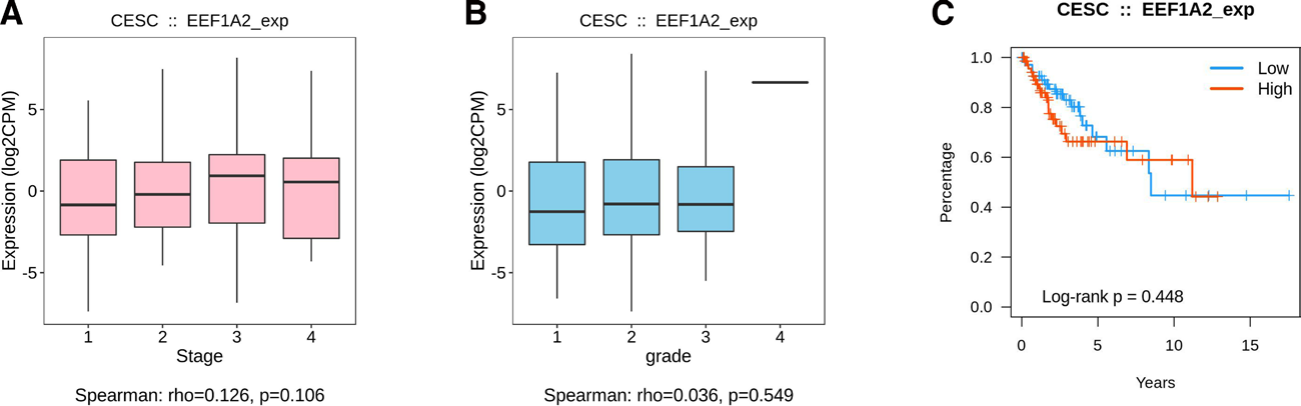
TISIDB database analysis. The relationship between the expression of *eEF1A2* protein and the clinical stage, pathological grade and survival of patients with cervical cancer. Note: (A) There was no statistical significance between the expression of *eEF1A2* protein and the clinical staging of cervical cancer (*P* = .106). (B) There was no statistical significance between the expression of *eEF1A2* protein and the pathological grading of cervical cancer (*P* = .549). (C) There was no statistical significance between the expression of *eEF1A2* protein and the survival of patients with cervical cancer (*P* = .448). *eEF1A2* = the expression of eukaryotic translation elongation factor 1 alpha 2. TISIDB = an integrated repository portal for tumor-immune system interactions.

Through an integrated repository portal for tumor-immune system interactions database analysis: There was no significant difference between the expression of *eEF1A2* protein and clinical stage in cervical cancer (*P* = .106) (Fig. 8A); There was no significant difference between the expression of *eEF1A2* protein and pathological grade in cervical cancer (*P* = .549) (Fig. 8B); There was no statistical significance between the expression of *eEF1A2* protein and the survival of patients with cervical cancer (*P* = .448) (Fig. 8C).

In summary, the expression of *eEF1A2* protein in cervical cancer has no statistical significance with the clinical stage, pathological grade and survival of patients by immunohistochemical database analysis (*P* > .05).

## 4. Discussion

The *eEF1A2* gene has attracted attention as an oncogene in different tumors.^[7]^ The *eEF1A2* is almost undetectable in normal breast tissue, whereas its expression is significantly increased in most breast tumors.^[8]^ Knockdown of *eEF1A2* by siRNA decreases phosphatidylinositol-4-kinase activity, suggesting that *eEF1A2* is a physiological regulator of phosphatidylinositol-4-kinase IIIβ signal transduction.^[9,10]^ The *eEF1A2* gene is involved in the activation of signal transducer and activator of transcription SATA3 and Akt, which may be used to inhibit cell proliferation, cell cycle progression, and cell apoptosis.^[11]^ The *eEF1A2* promotes the invasion of pancreatic cancer cells by upregulating the expression of MMP-9 through Akt activation.^[12]^ The *eEF1A2* promotes tumor formation in nude mice, and *eEF1A2* is expressed at high levels in some cases of multiple myeloma (a human plasma cell tumor). The *eEF1A2* may contribute to the development and progression of plasma cell tumors in mice and humans.^[13]^ Copy number variation is a region in the genome with different integer copy numbers. Copy number variants include the amplification and deletion of DNA sequences. copy number variants are an important source of genetic diversity^[14]^; they are the main source of variation between individuals and are an underlying factor in human evolution and a host of diseases, including mental illness, developmental disorders, and cancer. The mechanisms that lead to copy number changes include non-homologous end connection, perturbation of DNA replication, and replication of discontinuous DNA fragments.^[15]^ Copy number changes of long intergenic non-coding RNAs (lincRNAs) are an important mechanism that disrupts the expression of lincRNAs. Differentially expressed lincRNAs can predict the prognosis of cancer.^[16]^

In this study, bioinformatics analysis showed that the expression of *eEF1A2* did not differ significantly between cervical cancer and normal cervical tissues, and the expression level of the *eEF1A2* gene was not related to the survival period of cervical cancer patients. Real-time fluorescent quantitative PCR was used to detect the copy number of the *eEF1A2* gene in cervical cancer tissues, chronic cervicitis tissues, and normal cervical tissues. The results showed no statistically significant difference in *eEF1A2* gene copy number between cervical cancer and chronic cervicitis, indicating that changes in the expression of *eEF1A2* in cervical cancer tissues may not be caused by changes in the copy number of this gene.

We used cBioPortal to analyze mutations of the *eEF1A2* gene in cervical cancer tissues, and the results showed that there were missense mutations and splice mutations of the *eEF1A2* gene in cervical cancer patients, suggesting that *eEF1A2* gene mutation may play a role in the occurrence of cervical cancer.

## Acknowledgements

We thank International Science Editing (http://www.internationalscienceediting.com) for editing this manuscript.

## Author contributions

**Conceptualization:** Zemin Pan, Haichen Long.

**Data curation:** Haichen Long.

**Formal analysis:** Haichen Long.

**Funding acquisition:** Weinan Zheng, Zemin Pan, Haichen Long.

**Investigation:** Fang Wang.

**Methodology:** Weinan Zheng, Fuyuan Jin, Fang Wang, Luyue Wang, Shaowei Fu, Haichen Long.

**Project administration:** Weinan Zheng, Zemin Pan, Fuyuan Jin, Fang Wang.

**Resources:** Weinan Zheng, Zemin Pan, Haichen Long.

**Supervision:** Zemin Pan, Haichen Long, Weinan Zheng.

**Validation:** Fuyuan Jin, Fang Wang.

**Visualization:** Haichen Long.

**Writing – original draft:** Zemin Pan.

**Writing – review & editing:** Haichen Long.
